# Oxidative Damage in Lymphocytes of Copper Smelter Workers Correlated to Higher Levels of Excreted Arsenic

**DOI:** 10.1155/2010/403830

**Published:** 2010-12-28

**Authors:** Jorge Escobar, Lorena Varela-Nallar, Claudio Coddou, Pablo Nelson, Kevin Maisey, Daniel Valdés, Alexis Aspee, Victoria Espinosa, Carlos Rozas, Margarita Montoya, Cristian Mandiola, Felipe E. Rodríguez, Claudio Acuña-Castillo, Alejandro Escobar, Ricardo Fernández, Hernán Diaz, Mario Sandoval, Mónica Imarai, Miguel Rios

**Affiliations:** ^1^Instituto de Química, Facultad de Ciencias, Pontificia Universidad Católica de Valparaíso (PUCV), Avenida Brasil 119, Casilla 4059 Valparaíso, Chile; ^2^Departamento de Biología, Universidad de Santiago de Chile (USACH), Alameda 3363, Correo 40, Casilla 33, 9170022 Santiago, Chile; ^3^Centro de Biotecnología Acuícola, Universidad de Santiago de Chile (USACH), Alameda 3363, Correo 40, Casilla 33, 9170022 Santiago, Chile; ^4^Departamento de Cs del Ambiente, Facultad de Química y Biología, Universidad de Santiago de Chile (USACH), Alameda 3363, Correo 40, Casilla 33, 9170022 Santiago, Chile; ^5^Escuela de Medicina, Facultad de Ciencias Médicas, Universidad de Santiago de Chile (USACH), Alameda 3363, Correo 40, Casilla 33, 9170022 Santiago, Chile; ^6^Facultad de Ciencias Biológicas y Facultad de Medicina, Universidad Andrés Bello, República 252, 8370134 Santiago, Chile

## Abstract

Arsenic has been associated with multiple harmful effects at the cellular level. Indirectly these defects could be related to impairment of the integrity of the immune system, in particular in lymphoid population. To characterize the effect of Arsenic on redox status on this population, copper smelter workers and arsenic unexposed donors were recruited for this study. We analyzed urine samples and lymphocyte enriched fractions from donors to determinate arsenic levels and lymphocyte proliferation. Moreover, we studied the presence of oxidative markers MDA, vitamin E and SOD activity in donor plasma. Here we demonstrated that in human beings exposed to high arsenic concentrations, lymphocyte MDA and arsenic urinary levels showed a positive correlation with SOD activity, and a negative correlation with vitamin E serum levels. Strikingly, lymphocytes from the arsenic exposed population respond to a polyclonal stimulator, phytohemaglutinin, with higher rates of thymidine incorporation than lymphocytes of a control population. As well, similar *in vitro* responses to arsenic were observed using a T cell line. Our results suggest that chronic human exposure to arsenic induces oxidative damage in lymphocytes and could be considered more relevant than evaluation of T cell surveillance.

## 1. Introduction

Transition metals are ubiquitous elements in the environment, usually present in very low quantities in soil, food, and water. However, there are places with a combination of high geological levels and associated economic activities that result in chronic exposure of human populations to toxic levels of these metals. Arsenic (As) is a transition metal that occurs naturally in most ecosystems in northern Chile (hydrogeological characteristic of the region), and its presence is enhanced by human activities. Intensive copper mining in the area has increased arsenic levels in soil and water, thus increasing the risk to human health [1]. Nevertheless, because of mining processes, and in particular smelting plants within the mines and around the city of Chuquicamata, high levels of arsenic in the air are directly affecting miners [2–4]. Studies carried out on the effects of arsenic on people in work places have been considered important in order to measure the occupational risk of mine workers [5]. For instance, numerous adverse effects have been attributed to chronic arsenic exposure, including the appearance of different types of cancer [6], similar to studies of copper smelter workers in the USA that have shown a relationship between As exposure and cancer [7]. Similarly, a retrospective study in northern Chile involving people in areas with high exposure to arsenic showed increased incidence of lung, skin, and bladder cancer [8]. 

At the molecular level, physiopathological effects related to arsenic toxicity appear to involve different mechanisms and intracellular targets. Oxidative stress is among the most documented mechanisms of As toxicity and carcinogenicity. Oxidative stress is the result of an imbalance between radical oxygen species (ROS) production and the antioxidant defense system. Antioxidant response is one of the most efficient cellular defense mechanisms. Numerous enzymatic and nonenzymatic factors participate in cell protection by clearing and scavenging ROS. Superoxide dismutase (SOD) and vitamin E are among the main enzymatic and nonenzymatic mechanisms, respectively, involved in antioxidant response to As. The major active compounds of oxidative stress and oxidative signalling induced by As are ROS, which are able to damage molecules like DNA, proteins, and membrane lipids, generating the activation of signalling pathways to carcinogenesis. In general, the most important targets for arsenic-containing compounds appear to be mitochondrial proteins with a deflection of electrons from the respiratory chain to generate ROS. As well, ROS might be produced by cytosolic enzymes with peroxidase activity or during the oxidation of As^(III)^ to As^(V)^ (reviewed in [9]). ROS production by As may result in an attack, not only against antioxidant defenses and DNA, but also against membrane phospholipids, which are very sensitive to oxidation, producing peroxyl radicals and then malondialdehyde (MDA). In this regard, the application of radical scavengers has revealed the involvement of As-induced ROS in the induction of lipid peroxidation, as well as in DNA damage [10].

The aim of this work was to determine whether arsenic-driven oxidative activity shows a correlation with potential changes in lymphocytes obtained from copper smelter workers. We determined *in vitro* that lymphocytes from a highly exposed population showed higher thymidine incorporation compared to the control population. Moreover, a positive correlation between arsenic urinary levels and lymphocyte MDA levels and SOD activity, as well as a negative correlation with vitamin E serum levels, was observed. Similarly, SOD showed increased activity in highly exposed individuals and a positive correlation with As in urine. Our results indicate that chronic As exposure induces oxidative damage in lymphocytes. 

## 2. Materials and Methods

### 2.1. Ethics

The present study was carried out in Antofagasta, a northern region of Chile, where miners are environmentally exposed to high levels of arsenic. Protocols were approved by the Ethics Committee of the University of Santiago, Chile. All subjects signed an informed consent form. 

#### 2.1.1. Volunteers

From 160 smelter workers (section converters), a group of 40 were recruited for this study, workers met the following requirements: nonsmokers, no cardiovascular problems, and no severe problems of overweight, but with sedentary habits (none of them practiced sports regularly). None of them showed problems of alcoholism or symptoms of chronic illness such as hypertension or diabetes. Volunteers had a mean age of 43 ± 6 years (range 29–57), height 171.3 ± 5.5 cm (range 1.60–1.81), weight 74.9 ± 6.2 Kg, and body mass index of 25.6 ± 1.9 (range 21.5–29). As a control, thirty-two men with a mean age of 40 ± 7 years unexposed to arsenic smelt were recruited from Antofagasta. The control subjects have been exposed to minimal concentrations of arsenic in drinking water, with undetectable As urinary levels. Concerning the samples, The first morning urine sample and other urine samples were collected in polypropylene specimen containers and stored at −20°C before use. Blood samples were taken using sealed ethylenediaminetetraacetic acid (EDTA) Vacutainer Pb-free test tubes. Samples were stored at 4°C for less than 8 h until cell separation. Lymphocyte-enriched fractions were obtained from blood samples using Ficoll-Histopaque gradient sedimentation according to the manufacturer's protocol (Sigma Chem. Co., St. Louis, MO).

### 2.2. Proliferation Assay

To asses lymphocyte proliferation a [^3^H]-TdR incorporation assay was carried out under standard conditions, as described previously [11]. Briefly, 1 × 10^5^ cells were seeded in 96 well plates and exposed simultaneously to sodium arsenite (10^−6^ M) and 0.5 *μ*g/ml PHA (Sigma Chem. Co., St. Louis, MO). Cells were incubated for 48 h and then pulsed with 2 *μ*Ci/well of [^3^H]-TdR (Amersham Life Science). After 24 h cells were recovered and lysed with trichloroacetic acid 10%. The incorporation of [^3^H]-TdR was measured by liquid scintillation (Beckman, LS 6500). Background proliferation was determined using nonstimulated cells and subtracted from total counts, Jurkat cells.

### 2.3. Determination of Arsenic Levels in Urine

Urine samples were thawed at room temperature, diluted 10-fold, and centrifuged at 3000 × g at 4°C for 30 minutes. The precipitate was discarded, and the supernatant was passed through a 0.45 *μ*m filter (Waters, GHP Arcordisc Minispike). Then supernatants were diluted to 10 ml, and quantization of total content of arsenic was performed using an atomic absorption spectrophotometer, equipped with a standard Varian air-acetylene flame atomizer, as described previously in [12, 13]. Detection was done using a system coupling flow injection analysis with hydride generation atomic absorption spectrometry (FIA/HGAAS), as described previously in [14].

### 2.4. Determination of Oxidative Markers

 MDA formation was used to quantify lipid peroxidation in tissues and was measured as thiobarbituric acid-reactive material. Five million cells were homogenized in 1.15% KCl buffer. Homogenates were then added to a reaction mixture consisting of 1.5 ml of 0.8% thiobarbituric acid, 2 ml of 8.1% (vol/vol) SDS, 1.5 ml of 20% (vol/vol) acetic acid (pH 3.5), and 6 ml of distilled H_2_O and heated at 90°C for 45 minutes. After cooling at room temperature, the samples were cleared by centrifugation at 10,000 rpm for 10 minutes, and absorbance was measured at 532 nm by using 1,1,3,3-tetramethoxypropane as an external standard. The level of lipid peroxides was expressed as nanomol MDA per milligram of protein. Vitamin E was determined using HPLC as described previously in [15]. Standard curves were prepared by chromatographing known amounts of *α*-tocopherol (Sigma Chemical Co). All solvents were HPLC grade and filtered through Millipore 0.22 *μ*m membranes prior to use. These were washed with 5 ml each of diethyl ether and methanol before use. Vitamin E was eluted in silica gel columns (Bond Elut SI #601313 Analytichem International) and detection was performed by HPLC with a diode array detector. SOD activity was determined using the method of Misra and Fridovich [16] based on the inhibition of epinephrine autoxidation to adrenochrome.

### 2.5. Statistical Analysis

The Pearson *R* value was determined to test statistical correlations between MDA, Vitamin E, SOD activity, and arsenic concentration in urine. The difference in urine As content between the highly exposed group and the low-exposure group was analyzed using the U-Mann Whitney test. The difference in ^3^H-thymidine incorporation in lymphocytes between the control and As-exposed workers was analyzed using an unpaired *t*-test with Welch's correction. The multiple lineal correlation analysis between the urine As concentration was the outcome variable and sample T cell proliferation; plasmatic MDA and vitamin E concentrations were the causal variables. The statistical analysis was performed using Graphpad prism 5.0 software. All the statistical analyses were performed with a significance value of *P* ≤ .05.

## 3. Results

### 3.1. Arsenic Effects in Lymphocytes of the Exposed Population

**Figure 1:**
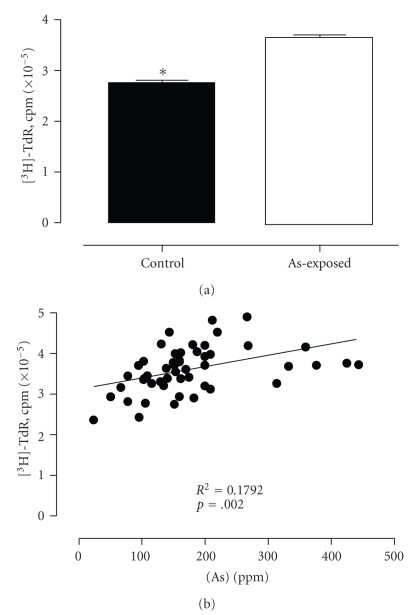
Comparative effect of arsenic exposure on the proliferation of lymphocytes isolated from low- and high-As-exposure human populations. Correlation of lymphocyte proliferation and urinary excretion of As from highly As-exposed population. (a) Proliferation was assessed by comparison of ^3^H-Thymidine incorporation ([^3^H]-TdR, cpm) in lymphocytes isolated from the low-As-exposure group (control group, *n* = 31) and from the high-As-exposure group (*n* = 50). Statistically significant differences were found between the two groups (U-Mann-Whitney test *P* = .0138). (b) Relationship between ^3^H-Thymidine incorporation and arsenic concentration in urine (ppm) of the highly exposed population (*n* = 50). Association between the two variables was determined by *R*-Pearson (*P* = .002).

Exposure to arsenic has been correlated with an increased prevalence to develop several diseases in human populations, including genotoxic damage and cancer. The main goal of this study was to evaluate the effects of arsenic on lymphocytes because damage to these cells could shed light on diseases developed in highly exposed populations. First, we evaluated [^3^H]-TdR incorporation in PHA-stimulated lymphocytes isolated from a highly exposed As group, as well as a control group of people from the same area. As shown in Figure 1(a), lymphocyte proliferation was observed in both groups of people studied. However, lymphocytes from the As-exposed population showed a higher rate of [^3^H]-TdR incorporation compared to those of the control group (3.32 ± 0.09 and 2.98 ± 0.47, resp., *P* = .014). Interestingly, there was a positive correlation (*P* = .002) between [^3^H]-TdR incorporation and arsenic salts present in urine collected over 24 h (Figure 1(b)). 

**Figure 2:**
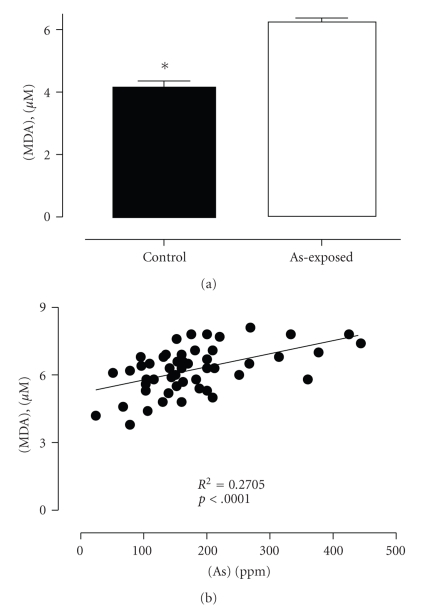
Comparative effect of arsenic exposure on lipid peroxidation in blood samples from low- and high-exposure human populations. (a) Comparison of MDA distribution profile in blood samples isolated from low-As-exposure (control, *n* = 31) and high-As-exposure (*n* = 50) human populations. Lipid peroxidation was assessed by plasma MDA concentration. The statistical analysis indicates significant differences between the two groups (test U-Mann-Whitney *P* < .0001). (b) Correlation between MDA concentration and urinary excretion of As in the high As-exposure population (*n* = 50). The associations between the two variables were determined by *R*-Pearson (*P* < .0001).

**Figure 3:**
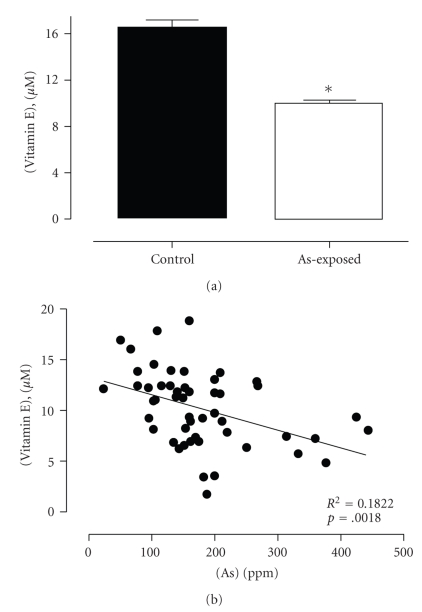
Comparative levels of vitamin E in plasma samples from low- and high-exposure human populations and correlation between plasma vitamin E concentration and urinary excretion of As from highly As-exposed population. (a) Vitamin E concentration profile in the plasma of the low-As-exposure group (*n* = 31) and the high-As-exposure group (*n* = 50). The statistical analysis indicates significant differences between the two groups (U-Mann-Whitney test *P* < .0001). (b) Relationship between plasma vitamin E concentration (*μ*M) and As concentration in urine (ppm) from the highly exposed population. The relationship between the two variables was determined by *R*-Pearson (*P* < .0018).

**Figure 4:**
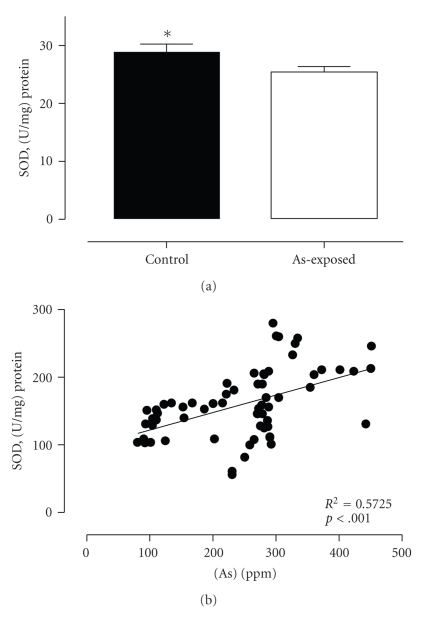
Comparative levels of SOD activity in plasma samples from low- and high-exposure human populations and correlation between SOD activity and urinary excretion of As from highly As-exposed population. (a) SOD activity in the plasma samples of the low-As-exposure group (*n* = 31) and high-As-exposure group (*n* = 50). Data are graphed as mean standard error (*P* < .0001). (b) Relationship between plasma SOD activity and As concentration in urine from highly exposed population (*P* < .0001). Statistical differences of SOD activity between the groups were established according to U-Mann-Whitney test.

We also evaluated the MDA, vitamin E, and plasmatic SOD activity on control and exposed populations to confirm oxidative stress and damage in lymphoid cells. As shown in Figure 2(a), MDA distribution in the two populations was different, being higher in the plasma of the highly exposed population compared to low-exposure population (6.231 ± 1.01 and 4.125 ± 1.31 *μ*M, resp., *P* < .0001). Analysis showed a significant and positive correlation between plasma MDA levels and arsenic in urine (*P* < .0001) (Figure 2(b)). If oxidative stress is related to an increase in oxidation of metabolites, response to arsenic injury might also be related to changes in plasmatic concentration of natural antioxidants. In support of this idea, we found that vitamin E plasma levels were lower in the highly exposed population than in the low-exposure population (9.765 ± 3.547 and 16.44 ± 4.332, resp., *P* < .0001) (Figure 3(a)). Moreover, we found an inverse correlation between vitamin E and urine concentration of arsenic (*P* < .0018) (Figure 3(b)). SOD activity measured from plasma samples in the control group was significantly higher than that in the high-As-exposure population (28.82 ± 12.39 and 25.29 ± 9.186, resp.) (Figure 4(a)), and this enzymatic activity correlates well with As concentration in urine from this population (*P* < .0001) (Figure 4(b)). Altogether, these results indicate that arsenic induces a significant increase of damage driven by free radicals in highly exposed people. To confirm a correlation between arsenic and the changes described in redox status, a multiple lineal correlation analysis was carried out. This correlation indicates that urine As concentration does not correlate with the sample T cell proliferation (*P* = .096) but does correlate with plasmatic MDA concentration (*P* = .004) and vitamin E concentration (*P* = .015). Additionally the standardized correlation coefficients indicate that an increase in urine As concentration correlates with an increase in plasma MDA concentration and decreased plasma vitamin E (standardized coefficients 0.374 and −0.294, resp.) indicating an increase in oxidative stress.

### 3.2. Arsenic Effects on the In Vitro Proliferation of Human Cells

**Figure 5:**
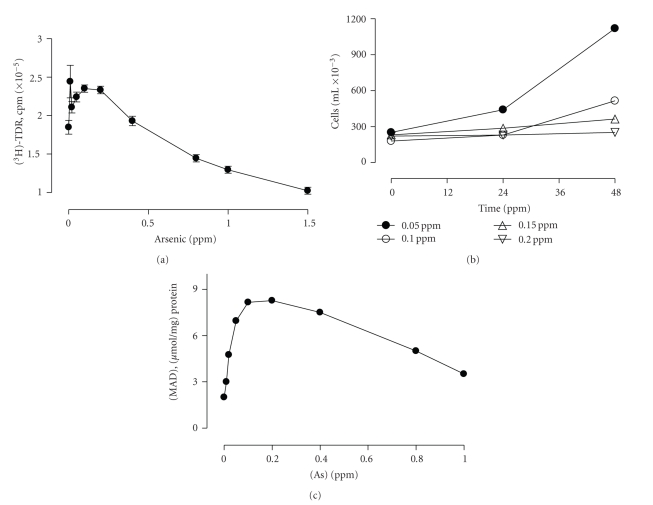
Effect of sodium arsenite on Jurkat cells. (a) Inhibition of proliferation of PHA-stimulated Jurkat cells by sodium arsenite. Cells (2 × 10^5^ cells/well) were incubated as indicated before, and proliferation was assessed by [^3^H]-TdR incorporation. Results are expressed in counts per minute (cpm) in the absence and presence of different sodium arsenite concentrations. (b) Evaluation of proliferation of cells (2 × 10^5^ cells/well) stimulated with 5 *μ*g/ml PHA for 48 h. Results are expressed as the number of cells obtained in the presence of different sodium arsenite concentrations in the culture media. (c) Lipid peroxidation in Jurkat cells induced by arsenic. Cells (2 × 10^5^ cells/well) were incubated with sodium arsenite for 48 h and the level of lipid peroxides was measured in the homogenates as MDA concentration.

Once the lymphocyte response and oxidative status of the highly exposed population had been characterized, we evaluated the effect of arsenic on T-lymphoma Jurkat cells. First, As (III) induced a reduction of thymidine incorporation during basal proliferation of human fibroblast cultures in a concentration-dependent form (data not shown). In contrast, a dual effect was observed in PHA-induced proliferation of Jurkat cells, that is, doses of 0.2 to 2 ppm of arsenic diminished [^3^H]-TdR incorporation, but lower doses of As (lower than 0.2 ppm) induced an increase (Figure 5(a)). As shown in Figure 5(b), the increase in [^3^H]-TdR incorporation did correlate to the total cell number in doses ranging from 0.05 to 0.2 ppm. Instead, a significant dose-dependent decrease of cell growth was observed until cell proliferation is completely arrested.

In order to evaluate oxidative stress-related response, lipid damage in response to arsenic treatment of Jurkat cells was studied by determining MDA levels, a marker of lipid peroxidation. Results showed that arsenic induces an increase in MDA in a dose-dependent manner, with a maximum increase observed at 0.2 ppm of arsenic (Figure 5(c)). Higher concentrations of the metal induced a decrease in MDA levels in Jurkat cells. Our results are in agreement with previous reports and support the hypothesis that low levels of arsenic induce mechanisms of DNA repair in cells because of oxidative cell damage.

## 4. Discussion

In this work we demonstrated that chronic arsenic exposure correlates with an increase in oxidative damage in lymphocytes and a downregulation of antioxidant mechanisms. Meanwhile, the mechanism of action of As in lymphocytes is not completely understood, the current consensus is that arsenic causes oxidative stress [17]. In this way, induction of several ROS best characterizes the effect of As exposure that causes cell damage [9]. Alteration in the antioxidant cell system has been reported in many pathologies involving metal-induced oxidative stress [18]. 

Here, we have demonstrated that humans chronically exposed to As also present changes in peripheral blood lymphocytes. Probably, the lymphocytes are trying to develop a response against the oxidative challenge, thus increasing antioxidant defences. In this respect, the literature is contradictory, showing that lymphocytes treated *in vitro* with arsenic exhibit a lower level of activity of some antioxidant enzymes, such as catalase, glutathione peroxidase, and SOD [19]. However, this study was conducted *in vitro* and with acute exposure. On the other hand, another work reported that As exposure induced significantly higher SOD activity *in vitro* [20]. A study conducted in Inner Mongolia, China, with humans exposed to highly contaminated drinking water, did not show significant differences in blood SOD activity [21]. However, the authors of this study compared the highly exposed population to a group with low exposure, the latter still having twice the As level as accepted level for drinking water by the World Health Organization. In light of our results, however, in humans chronically exposed to As-contaminated air SOD activity is induced in a direct relationship to As excreted, and probably to the levels of As in plasma. As well, the response to As-induced damage can include modification of cellular antioxidant uptake or alteration in the activity or its oxidation. The use of antioxidants like vitamin E has been described as an efficient strategy to protect cells from As-induced oxidative damage [22]. However, until now no examination of the levels of vitamin E in blood in an exposed population has been described. We found a significant decrease in vitamin E, which is directly related to the excreted As levels.

Strikingly, there is controversial evidence about the effect of arsenic on lymphocyte proliferation, *in vitro* challenge results in inhibiting lymphocyte proliferation associated with decreased thymidine incorporation. *In vitro *studies indicate that arsenic-delayed proliferation of T-lymphocytes and modified DNA synthesis occur in a biphasic dose-dependent manner [23, 24], high doses of As inhibit PHA-induced T cell proliferation [23, 25, 26], while lower arsenic doses used *in vitro* increase lymphocyte proliferation induced by PHA, in agreement with our results *in vitro* [24, 27]. On the other hand, while we determined that lymphocytes of exposed workers show increased thymidine incorporation, the literature reports decreased lymphocyte proliferation in a murine model [28] and decreases or no changes in lymphocytes of people exposed to arsenic in drinking water in Mexico. A report shows that adults chronically exposed to high levels of As had lymphocytes with a lower average generation time compared to that of individuals with less exposure to the metal. Unfortunately, the number of cases analysed was low and did not permit comparison with confidence [29]. Other cases of adults in Mexico did not show changes in the replicative index of lymphocyte obtained from individuals with chronic exposure to As via drinking water [30]. A more recent study suggests that lymphocytes from children exposed to As show reduced cell mitogenic response to PHA [31]. All of these observations are in disagreement with our results. Unfortunately, we do not have explanations for these differences, except that the doses present in the workers in our study could have been lower than those observed in people exposed to arsenic from water or could be related to regionally specific differences and other metals present in the air, mines, or in the water in Mexico. However, it has been repeatedly suggested and demonstrated that the exposure of cells to low levels of ROS induces division and proliferation (for review see [32]). It has been widely documented that the use of antioxidant or ROS scavengers, as discussed above, protects against As-induced damage. In this respect, we can speculate that the induction of cell proliferation can be found at levels of As concentration at which the toxicity is mainly due to ROS generation.

A retrospective study in the same geographic area as our study recently described an increased prevalence of cancer compared to other parts of Chile [8]. Similarly, other studies reported that chronic inhalation of arsenic is related to leukaemia and lung cancer and urinary excretion of arsenic metabolites [33–35]. In the USA, arsenic in drinking water has been correlated with several diseases, such as cancer, cardiovascular disease, and diabetes [36–41]. The role of arsenic in cancer induction has been not clearly established, but it is supported by the idea of generating genotoxicity. Our results support the idea that the prevalence of cancer could be related both to changes in oxidative stress, which can damage the genome, and to altered lymphocyte activity or response. In this way, there is substantial evidence supporting the latter idea, besides the changes described in the proliferative response of lymphocytes. For instance, *in vitro *studies indicate that As inhibits IL-2 and IL-4 secretion and CD25 expression [23, 25, 26]. As well, it has been reported in India and in the other countries, where human populations have been exposed to arsenic in drinking water, that peripheral lymphocytes show an increased frequency of sister chromatid exchanges [42–44]. Moreover, Yu and coworkers showed that PBMC from patients with arsenic-induced Bowen's disease had a decreased percentage of T cells, particularly the T helper subpopulation [45]. Other important and recent evidence implies that lower *in vitro *concentrations of arsenic increase the number and function of T regulatory lymphocytes [46]. Altogether, the damage induced in proliferative cells, as well as the noxious effects on lymphocyte functions, that is, induction of anti-inflammatory and T regulatory lymphocytes at low concentrations, supports a different role of arsenic, not only as a possible inductor of cancer but also linked to impaired lymphocyte function. However, we cannot exclude other transition metals that are also capable of causing oxidative degradation of lymphocytes.

##  Conflict of Interests

The authors declare no conflict of interest. Protocols were approved by the Ethics Committee of the University of Santiago, Chile. All subjects signed an informed consent form.

## Abbreviations

As: Arsenic
ROS: Radical oxygen species
SOD: Superoxide dismutase
MDA: Malondialdehyde.
